# Can We Predict Adductor Strain? A Predictive Analysis of a Major League Soccer (MLS) Cohort Spanning from 2019 to 2022

**DOI:** 10.3390/jfmk11010108

**Published:** 2026-03-05

**Authors:** Rebecca Davis, Benjamin C. Brewer, Martha Hall, Jill S. Higginson

**Affiliations:** 1 Biomechanics and Movement Science, University of Delaware, Newark, DE 19716, USA; 2 Mechanical Engineering Department, University of Delaware, Newark, DE 19716, USA; 3 Biostatistics, Department of Epidemiology, University of Delaware, Newark, DE 19716, USA

**Keywords:** adductor strain, predictive modeling, professional soccer, MLS, injury risk, model framework

## Abstract

**Background:** Despite the high prevalence of adductor injury in soccer, there is limited injury-specific predictive modeling to identify common risk factors. The objective of this study was to create an adductor strain prediction model utilizing injury, game, and performance data collected from a cohort of professional Major League Soccer (MLS) players. **Methods:** We identified potential risk factors for soft tissue, non-contact adductor strain using a predictive machine learning model framework. Performance and injury data were collected between the 2019 to 2022 seasons of one professional MLS team. We utilized Random Forest (RF) machine learning models with Synthetic Minority Oversampling (SMOTE) to predict soft tissue, non-contact adductor strain injury amongst the cohort. Features chosen to be implemented in the model included injury, game, and performance data. **Results:** From the four models constructed in this study, the best performing model included Catapult Global Position System (GPS)/Internal Measurement Unit (IMU), strength, injury, and game data using a weekly structure determined by F1 score. Multiple models indicated that not having a previous injury lowers the odds of a future injury in the following week or month. Forwards had greater odds of injury whereas defenders had lower odds of injury. Greater hamstring max force lowered odds of injury whereas a greater amount of change of direction efforts increased the odds of injury in the following week or month. Adductor-to-abductor max strength ratio showed conflicting results regarding the odds of future injury. **Conclusions:** Through the utilization of RF and SMOTE, we were able to successfully predict adductor injuries in an MLS cohort utilizing injury, game, and performance metrics. Validation in a larger cohort would be highly recommended before utilizing the findings of this study in the design of injury prevention protocols.

## 1. Introduction

Adductor strains are one of the most common injuries endured by soccer players at all levels [1,2,3,4]. Depending on the grade of strain, rehabilitation, and/or the surgical procedures undergone, athletes can experience anywhere from a single day to an entire season out of play, however, the median time away from play ranges from 14 to 22 days [4,5]. Adductor reinjuries have been found to occur in 6 to 21% of injured adult male soccer players ranging in competitive levels [5,6,7] and have been found to have a significantly longer recovery time compared to other lower extremity injuries [5]. Due to the impact this can have on a professional athlete’s career and team, the scientific community has made it a priority to identify risk factors for such injuries in an effort to reduce injury risk. A proactive approach to injury mitigation could save players days, weeks, or months of time away from play [8]. Predicting injuries, however, is not straightforward and some argue is nearly impossible as current methods are not sufficient to make these predictions in a clinical setting [9,10,11]. Arnason et al. as well as other researchers have found through logistic regression and further correlation analyses that previous injury is a significant risk factor for future injury [12,13,14,15,16,17,18]. Adductor injuries and more broadly lower extremity soft tissue injuries can occur from dynamic and multifactorial changes throughout the course of an athlete’s career [17,19,20,21]. Due to the nature of such injuries, machine learning models have been utilized to account for the complex environments in which an injury occurs. Machine learning models and techniques have been utilized to predict injury among athletes [11,19,22,23,24,25,26,27,28,29,30,31,32,33,34,35,36,37,38]. The predominant machine learning algorithms that have been used to identify injury risk in individuals include logistic and linear regressions, decision trees, random forests (RF), boosting, k-nearest neighbors, artificial neural networks, and support vector machines. Random Forest has been a promising algorithm due to its precision in injury prediction [24,27,30,31,33,38]. RF is able to be trained on smaller sets of data when compared to other techniques such as neural networks. Additionally, RF can account for nonlinear relationships, and while it is considered a black box model that can be difficult to interpret, an understanding of influencing features through variable importance (VIMP) and the directionality of the variables through odds ratios from logistic regression can be obtained [39].

A common problem with injury prediction is the small observation size in the injured class. Techniques such as Synthetic Minority Oversample (SMOTE) [40], and random over and under sampling are generally used to address the issue of data imbalance. In previous injury prediction studies, SMOTE has been utilized to create synthetic injury data to extrapolate from limited data points and has shown promising results in the ability to predict injury [19,24,29,37,41]. However, this is not always the case [25,36]; for example, Carey et al. found in Australian football with a hamstring injury prediction model, using workload variables from wearable sensors such as total distance, running speeds, rate of perceived exertion, and player load, that SMOTE pre-processing showed no such increase in prediction performance defined by the area under the receiver operating characteristic curve (AUC) which explains how well the model was able to separate between the injured and non-injured classes [36]. While SMOTE showed no increase in prediction performance, their injury specific hamstring model resulted in improved model performance compared to their models with inclusion of all non-contact injuries [36]. In contrast, Robles-Palazón et al. assessed model performance with and without class balancing techniques to predict soft tissue, lower extremity injuries in youth soccer players and found multiple techniques such as SMOTE and random undersampling (RUS) increased the AUC in all employed machine learning models [37]. While predictive models for soft tissue lower extremity injury have been constructed, an adductor strain-specific predictive model has yet to be reported in a group of professional soccer players within Major League Soccer (MLS) over multiple seasons.

The objective of this study was to build a soft tissue non-contact adductor strain predictive model utilizing data from a single MLS team over multiple seasons to gain greater insight into the multifactorial relationships best able to predict injury. RF models were constructed and data imbalance was addressed with the utilization of SMOTE. We utilized common input features typically collected at professional soccer clubs such as adductor and abductor strength, inertial measurement unit (IMU) calculated accelerations, decelerations, and change of direction (COD) movements during play, past adductor injury history, and descriptive factors such as when they played in a game and position. With this information, we aim to gain insight into variables affecting adductor injury risk.

## 2. Material and Methods

### 2.1. Study Design and Participants

Participants in this study were all male athletes contracted to a single US MLS team throughout the years 2019–2022. This team was comprised of professional soccer players who are at the highest level of soccer performance in the MLS and within this respective team regarded as the first team. No data was used regarding players in the second, academy, or other organized teams with which players may have been previously contracted, however, if a first team player played in a second team game on occasion, that player’s second team game data would be included in this study. Models were structured to satisfy a weekly and monthly predictive future time frame. Weekly data was collected and used to predict an athlete’s risk of injury in the following week while monthly data was compiled to predict an athlete’s risk of injury in the following month. University of Delaware IRB exemption was given due to the retrospective and deidentified data used within this study. In addition, MLS M-Marc authorization was given to obtain this data and conduct this study.

### 2.2. Procedures and Instruments

#### 2.2.1. Injury Documentation

Injury logs were reviewed from the years 2019 to 2022. These injury logs were recorded by the athletic trainers and physical therapists employed by the team. Injury logs were created at the time of injury. Information such as injury type, mechanism of injury (MOI), location of injury, field position, and game or practice environment were recorded. For the purposes of this study, we only included soft tissue adductor injuries with non-contact injury mechanisms as this removes the unpredictable nature of external forces acting on the body such as a collision with another player or object. Features from the injury log that were used in this analysis included position type (forward, midfielder, defender, or goalkeeper) and time since adductor injury defined as 0 to 2 months, 12 months or more, and never injured.

#### 2.2.2. Game and Daily Monitoring Systems

Game data was collected through Opta Tracking (Opta Tracking, Stats Perform, London, UK) which tracks which players competed in a game as well as the minutes played for each person in each game. This information was utilized to quantify in-game exposure time for athletes. We included both first and second-team games as many of the contracted first team players would also play in second-team games if they did not have enough in-game exposure time for the week. The second team is the next level below the highest level of professional sport at this club. Opponent and team strategy can lead to different demands between these two levels, however, these differences were not investigated in this study.

Position sensors were worn in a vest with the sensor located between the scapular region (Vector, Catapult, Melbourne, Australia). These sensors utilized both an inertial measurement unit (IMU) and global positioning system (GPS) to measure metrics such as distance run, velocity, acceleration, deceleration, and COD. Athletes wore these sensors during practice and game events between the 2019 to 2022 seasons. Data was stored on the sensors and downloaded to the Catapult platform after practice or game events by the team’s Sports Scientist. COD is categorized in the direction of an athlete’s dominant and non-dominant side defined by the leg they kick with. Catapult has different acceleration metrics labeled as Inertial Movement Analysis (IMA) acceleration and 2nd generation acceleration efforts. The IMA and 2nd generation acceleration efforts are calculated differently within the Catapult system. We found that these metrics were not highly correlated with each other and since both metrics were recorded with Catapult sensors, we included both. However, 2nd generation acceleration efforts had only been recorded from 2020 to 2022 for this cohort and will only be included in select models. In both metrics, thresholds were defined for an acceleration effort. In IMA, this was defined as any qualifying effort over 3.5 G of force and for 2nd generation, any qualifying effort over 3 m/s was included. This was done to avoid any potential misclassification of low/mid acceleration efforts and to assess acceleration efforts in a higher-intensity state. Deceleration efforts were calculated using the IMA corresponding inverse threshold.

Exponential weighted moving averages (EWMA) were calculated for the acceleration, deceleration, and COD efforts and were included as input features to the models. Equation (1) outlines an example of how EWMA is calculated for acceleration efforts.

(1) $${EWMA}_{today}=\left(\frac{2}{N+1}\right)\times {Accel}_{eff,today}+\left(1-\frac{2}{N+1}\right)\times {EWMA}_{today-1day}+\cdots \;,$$

This allows greater weight to be given to the most recent collected data point where points taken farther away in time become less influential. This has been found to be a more accurate depiction of the average workload over time rather than a simple averaged effort calculation [42,43]. We constructed this on a weekly and monthly basis for the two model frameworks. EWMA was calculated on a daily basis with regard to the previous 28-day period (*n* = 28) and then those values were averaged for defined week and month time frames.

#### 2.2.3. Strength Assessments

Adductor, abductor, (ForceFrame, VALD Performance, Newstead, Australia) and hamstring (Nordic, NordBord, VALD Performance, Newstead, Australia) strength tests were conducted throughout the 2021 and 2022 seasons. The ForceFrame utilizes uniaxial load cells integrated into pads that can be adjusted for differing isometric assessments and training and has been found to be a reliable and valid testing method for hip strength [44]. Adductor and abductor isometric testing were conducted approximately every eight weeks throughout the 2021 and 2022 seasons. The athlete would be in a supine position with knee flexion at 45 degrees. They were instructed to give a maximal exertion with a 30-second break in between for 3 to 5 repetitions. Athletes were instructed to squeeze onto the pads for adductor strength testing and push for abduction strength testing bilaterally. From this test, we extracted peak force (N) production in the dominant and non-dominant limbs from adduction and abduction as well as interlimb imbalance and ad/abductor imbalance ratio.

Similarly, Nordic utilizes two load cells to measure force and uses a platform to mark the center of knee location to calculate torque with a defined moment arm. This test has shown fair to high inter and intrasession reliability [45,46]. The hamstring Nordic strength assessments were conducted approximately every 6–8 weeks throughout the 2021 and 2022 seasons. Athletes were instructed to kneel on the Nordic platform and keep their hips forward with arms crossed on their chest. Their ankles were secured by hooks in a 90-degree position relative to their lower legs. They were instructed to lean forward under tension for as long as possible before they needed to catch themselves from falling for 1 to 4 repetitions. From these tests, we reported maximum force (N) and interlimb maximum force imbalance.

### 2.3. Data Analysis

If individuals did not have recorded data from the Catapult sensors, they were logged as having zero acceleration, deceleration, and COD efforts for that day. Efforts were calculated as a summed number of total efforts per week or month depending on the model. For a given participant, strength assessment values were left as missing until the first recorded assessment, after which that value was repeated until a new assessment value was reached. If there were multiple of the same test taken within a given week or month, the values were averaged together to get the respective weekly or monthly strength value.

A Random Forest (RF) algorithm was used to determine if the available set of performance metrics could identify adductor strain injury outcomes. RF was selected due to its ability to robustly iterate through thousands of decision trees to assess the features’ ability to predict injury. RF does not need large datasets to train the model in comparison to neural networks for example which may perform better but tend to be more computationally extensive. The utilization of RF also allows us to accurately assess categorical and continuous variables appropriately over time while taking into consideration non-linear and linear relationships that may be present. All features included in the model were determined through assessment of collinearity and selective reduction; namely, pairs of variables with an observed correlation coefficient greater than 0.60 were represented by a single variable in the pair. The single variable was chosen based on its correlation to adductor injury. We set up multiple weekly and monthly models utilizing game, injury, and performance data to assess the model’s prediction error and identify which variables had the greatest variable importance (VIMP), a measure that gives a variable’s relative importance in producing an accurate prediction; variables with higher VIMPs are more instrumental in producing accurate predictions. Four models were created: Models containing Catapult, game, and injury data from 2019 to 2022 were denoted as Catapult Weekly (CW) and Catapult Monthly (CM). Since consistent strength assessments were only collected in 2021 and 2022 seasons, weekly and monthly models were created with a reduced set of Catapult, game, injury, and strength data and denoted as Catapult Strength Weekly (CSW) and Catapult Strength Monthly (CSM).

SMOTE techniques utilizing a k-nearest neighbors approach to create ten times the synthetic data points from the injured population were employed [40]. We also reduced the non-injured data points via RUS, maintaining a representative group in the process. For every model, in an attempt to avoid too aggressively oversampling, we balanced the data set to ensure that 25% was comprised of injury data points and 75% were comprised of no injury data points. Previous prediction models have employed SMOTE techniques to predict sports injury and mortality rates in differing populations. To balance the data in these studies, research groups have reported upsampling the minority class to 40% of the data set and randomly undersampling the majority class to 60% of the total data set [19,37]. For this analysis, we selected a more conservative 25 to 75% split for minority and majority class sample sizes, respectively.

Initial RFs were run with a defined set of predictor variables (Table 1) through practitioner knowledge of adductor injury and the aforementioned assessment of collinearity. The prediction error was recorded for both the non-injured and injured populations. Two different procedures for testing the model were utilized: (1) Train each model on 2019–2021 data and use 2022 as testing data, and (2) Include all years of data collection, train the model with 80% of the dataset, and test the model on the remaining 20%. In both cases, we then measured variable importance through VIMPs and carried out bootstrapping, a statistical technique featuring repeated re-sampling with replacement, in order to establish the significance of the VIMPs. F1 scores were calculated to assess model accuracy as well as classification error of the injured and non-injured classes.

Odds ratios were obtained through logistic regression to assess the directionality (i.e., protective or risk-increasing) of the variables. In some cases, the corresponding logistic regression model did not converge; in these cases, odds ratios are not provided. This phenomenon is generally seen for categorical variables where the crosstabulation of injury and the categorical variable feature some cells having a count of 0. RF model performance is reported through a confusion matrix where a total misclassification percentage is given for injured and non-injured predictions. From this, we also obtain percent error for model-predicted injury and non-injury.

## 3. Results

From 2019 to 2022, 53 athletes were contracted to the MLS first team. Within this group, eight athletes endured an adductor injury. A total of nine adductor injuries were recorded during this period. Each model had a different number of injuries and athletes recorded due to the variables used and the timing in which the injuries occurred. This information in conjunction with the data points present before and after the utilization of SMOTE and RUS can be seen in Table 2.

Due to the limited sample size of injuries within this cohort, as stated previously, we felt it was necessary to (1) test the model on the 2022 season data as well as (2) utilize the full data set, training on 80% of the data and testing on the remaining 20% which allows the model to be trained on a large subset of the data and tested on unseen data with partial reference to the Pareto principle. Only two adductor injuries were recorded during the 2022 season. The predictive models were not successful in predicting either of these injuries, showing that validation strategy (1) did not demonstrate adequate model predictive performance (i.e., 100% error rate of the injured class). Conversely, validation strategy (2) demonstrated much stronger model performance.

Weekly and monthly structured models were created utilizing player characteristics and performance metrics collected. Results of the RF and logistic regression (LR) of CW and CM models are shown in Table 3; CSW and CSM model results are shown in Table 4.

The CW model included 14 variables with position as a defender (P_def_) and no prior injuries (PI_never_) identified as significant variables related to decreased odds of adductor injury (*p* < 0.05, Table 3, F1 score (0.942)). Defenders have 82.4% lower odds of adductor injury in the following week compared to those who were not defenders. Individuals who have never had a previous adductor injury have 80.7% lower odds of getting a future adductor strain in the following week as compared to individuals who have had a previous adductor injury.

The CM model included the same 14 variables as CW, however COD efforts (COD_eff_) and history of ever having a previous adductor injury (PI_never_) emerged as significant features (*p* < 0.05, Table 3, F1 score (0.932)). Each additional COD effort in a month was associated with a 5.7% increased odds of injury in the following month. Individuals with no previous adductor injury had 90.6% lower odds of enduring a future adductor strain in the following month when compared to those with a previous adductor strain. The logistic regression was not able to converge on the variable, P_def_, as not all categorical options are present as stated in the statistical approach.

From the CSW, significant variables included P_for_, P_mid_, P_def_, COD_eff_, Ab_maxf_, Ad_maxf_, Ham_maxf_, and Ratio_adab_ (*p* < 0.05, Table 4, F1 score (0.969)). In the CSW model, forwards had 9.173 times the odds of future adductor injury in the following week compared to non-forwards. Defenders and midfielders exhibited the opposite effect. Defenders had 54.2% and midfielders had 63.7% lower odds of adductor injury in the following week compared to non-defenders and non-midfielders. Individuals with greater hamstring max force were found to have lower odds (0.6% per 1 N of force) of getting a future adductor strain in the following week. Each unit increase in adductor-to-abductor max force ratio was associated with 86.2% lower odds of adductor injury in the following week. Greater abductor (0.1% per 1 N force) and adductor (0.2% per 1 N force) max force generation was related to greater odds of adductor injury in the following week. Finally, individuals who had more COD efforts had greater odds (0.4% per 1 COD effort) of getting an adductor strain in the following week.

Significant variables identified in the CSM included Accel2_eff_, P_for_, PI_never_, Ham_maxf_, and Ratio_adab_ (*p* < 0.05, Table 4, F1 score (0.962)). In the CSM model, forwards had 19.998 times the odds of a future adductor injury in the following month compared to non-forwards. Hamstring max force showed the same influence as seen in the weekly model, where individuals with greater hamstring max force were found to have lower odds (0.6% per 1 N of force) of getting a future adductor strain in the following month. The ratio of max adductor to max abductor force exhibited differences in findings between the weekly and monthly models. In the monthly model, each unit increase in adductor-to-abductor max force ratio was associated with 16.956 times the odds of a future adductor injury in the following month; this is the opposite direction seen in the weekly model. Those with no previous adductor injury had 54.2% lower odds of future adductor injury in the following month compared to those with a previous injury. Generation 2 acceleration efforts showed increased odds (5.3% per 1 acceleration effort) of adductor injury in the following month.

Between all models, the significant variables that predict adductor injury most often included PI_never_, Ratio_adab_, Ham_maxf_, COD_eff_, P_for_, P_def_. Other variables showed significance and should be taken into consideration, however, as seen, results vary between the weekly and monthly predictive frameworks.

Other variables such as cumulative minutes played in a game and number of games played did not emerge as significant influencers in any models. EWMA of acceleration, deceleration, and COD as well as IMA efforts showed no significance. Injuries 0 to 2 months prior and injuries that occurred 12+ months ago did not show significance. The interlimb imbalance between the adductors, abductors, and hamstrings were not significant predictors of adductor injury.

Overall, we see that the CSW and CSM models had the lowest prediction error for the injured population (Table 5). The CW and CM models also performed well, showing less than 12% misclassification of the injured population. CSM had the lowest injury misclassification of 5% however it did misclassify a small % of the non-injured class as injured. The CSW, however, did not misclassify any non-injured data points and had a 6% error in predicting the injured class. The model that performed best overall was the CSW model with a 1.5% error with the CSM, CW, and CM models following closely behind.

## 4. Discussion

Due to the prevalence of adductor injury in the soccer population, we aimed to create a predictive model that identifies characteristics that may put an athlete at higher risk of injury [1,2,3,4,5,6,7]. This study was conducted on a single men’s professional team within the MLS utilizing data from the 2019 to 2022 seasons. From this study, we were able to create predictive injury models for the following week and month that were successful in classifying injury and non-injury occurrences within this cohort. We utilized player position, game, Catapult, and strength data to extract features of importance in the models. The findings show that position type, history of previous adductor injury, COD and 2nd generation acceleration efforts, maximum force production of the adductors, abductors, and hamstrings, and the adductor-to-abductor ratio were identified as significant variables in at least one of the four models created. While these findings have the potential for minimal classification error of the healthy population, or false positives, it is important to note that error in classifying the injured population, or false negatives, are present and should be taken into consideration when implementing these findings in practice. Acceptable error in classifying the injured and non-injured class should be determined prior to the application of such models.

The adductor muscle group is a complex group within the core musculature that has multiple functions including adduction, internal rotation, flexion, and extension of the hip as well as joint stability in the frontal plane [47,48]. Due to this, we assumed the model best able to predict injury would take into consideration multiple risk factors associated with injury. We utilized tests that assessed strength in the planes of motion in which the adductors act and assessed quantities of movements where the adductor can be put in environments of great strain such as COD, acceleration, and deceleration movements. Breaking down the findings between each model shows unique variable importance. In the CW model, we found that being a defender and never having a previous adductor injury lowers the odds of future injury in the following week. In the CM model, we observed that never having a previous adductor injury lowers the odds of getting an adductor strain in the following month. As stated in the results, previous injuries 0 to 2 months and 12+ months prior and not show significance which could be due to insufficient reinjury data within the data set. In addition, a greater amount of COD efforts is related to higher odds of injury in the following month.

The CSW model shows midfielders, defenders, greater adductor to abductor max force ratio, and greater hamstring max force generation are related to lower odds of adductor injury in the following week. Forwards, greater COD efforts, greater abduction max force, and greater adduction max force were related to greater odds of adductor injury in the following week. In the CSM model we see no previous adductor injury and greater hamstring max force generation is related to lower odds of adductor injury in the following month whereas forwards, greater generation 2 acceleration efforts, and greater ad/abductor max force ratio are related to higher odds of injury in the following month.

We see forwards tend to have greater odds of injury whereas defenders have lessened odds compared to other position types. This could be explained by the unique demands pertaining to acceleration, top running speeds, distance traveled, and strain that each position type experiences. It has been found that forwards and midfielders tend to have most amount of acceleration efforts on average, and high-intensity distance covered in match play whereas defenders tend to have lower high-intensity distances covered and average number of acceleration movements [49,50,51]. A team’s formation patterns on the field can influence these demands so internal position-type workload comparison may be beneficial. High-intensity movements such as acceleration, deceleration, and change of direction can put the adductors under greater strain and may help explain the differences we see in position type injury risk.

In addition, we found that athletes with no previous history of adductor injury had between 54.2% to 90.6% lower odds of getting injured in the following week or month compared to individuals who had a previous injury. Following muscle injury, structural changes to the musculature and extensive scar tissue can occur which can alter the functionality of the muscle and can lead to reduced strength, greater stiffness, and the potential for reinjury due to the inability to sustain contractile forces [52,53,54,55,56,57,58,59]. This could explain why individuals with no history of adductor injury have lower odds of future injury compared to those with previous injury. The findings from this study provide further evidence that previous injury is a well-defined risk factor of future adductor injury [12,13,14,15,16,17,18]. Hamilton et al. highlighted that there are modifiable and non-modifiable factors that could influence one’s risk of re-injury [60]. Appropriate rehabilitation can address modifiable factors such as structural issues of the muscle, loss of function, imbalance, and compensatory patterns through other muscle groups. On the other hand, previous injury could be an indicator of non-modifiable intrapersonal characteristics such as position type, age, or hip morphologies that could increase one’s risk of injury. It is important to acknowledge and consider non-modifiable factors while addressing modifiable risk factors and appropriately monitoring adductor function for return to sport.

A greater number of COD efforts in game and practice tend to increase the odds of injury whereas a greater Nordic hamstring max force generation has been shown to result in lower odds of adductor injury. When interpreted, we see that the accumulation of COD efforts throughout a week and monthly period can result in much greater odds of injury. As seen in Table 4, for every 1 COD effort, we saw a 0.4% increase in odds of injury in the future week. For example, if player A did 10 COD efforts and player B did 0 efforts for one week, player A would have 4% greater odds of adductor injury in the following week. It is important to note how this can scale when some athletes may experience over 100 efforts in a week. During a COD movement, great strain is put on the adductor musculature as one experiences an adduction movement against an abduction force. These findings support that a greater number of COD efforts is related to adductor injury risk. Consequently, monitoring for greater efforts among individuals for a given week or month could be advised.

The ratio of the adduction-to-abduction max force values showed differing results between the monthly and weekly models. We found that a greater adductor-to-abductor max force ratio would lead to greater odds of injury risk in the following month. However, in the weekly model, individuals with stronger adductor max force compared to abductor max force had lower odds of adductor injury in the following week. The testing windows in this study can introduce repeated measures week-to-week and month-to-month leading to reduced accuracy in acute interpretations. Results suggest that greater adductor max isometric force capability relative to the abductors may have a protective effect on immediate demands experienced by an athlete in the following week, hence reducing risk of injury. However, athletes who maintain such a strength imbalance could be hypothesized to be at a higher risk of adductor injury in subsequent months due to chronic adaptations of greater adductor to abductor max force production. This could influence an athlete’s movement strategies and decrease pelvic stability, placing greater strain on the adductors and stabilizing structure during repetitive high force generating movements. Further investigation as to the differences seen in these models and physiological time-dependent responses need to be investigated. There has been supporting evidence found that an increased abductor-to-adductor strength ratio is a risk factor for adductor injury [17,61].

Max hamstring strength was found to be a significant input variable within the prediction models. The functions of the adductors, specifically the adductor magnus, may give context as to why this variable is significant in predicting injury. The adductor magnus is comprised of two sections, the pubofemoral and ischiocondylar portions [62]. Due to the similarities in innervation and actions of the ischiocondylar portion to the hamstrings as a hip extensor, it brings up the question of whether we can consider hamstring assessments such as the Nordic to be a good assessor for adductor functionality as well. In addition, the Nordic movement relies on stabilization of the trunk and pelvis [63]. While greater all-around strength from the adductors and other pelvic and trunk stabilizers may help sustain the Nordic movement longer, it is not known if it would directly affect max force generation. This would require observation of the muscle activity during this specific movement and potential compensatory patterns that could be present in individuals. However, the adductors play a key role in the stabilization of the pelvis [48,62], which could explain why greater force generation in a functional eccentric movement that relies on pelvic stabilization to assess hamstring strength could be related to lowered odds of adductor injury risk. We found with a 1 N increase in hamstring max force, a person would have 0.6% lower odds of future adductor injury in the following week and month. Since the maximum force is scaled in the hundreds of Newtons, we can see how this has the potential to influence a person’s risk of future injury.

In most instances, it is better to have a false negative of the non-injured population if that means having better precision in classifying the injured group. This may lead one to favor the CSM model with a lower % error of the injured class classification even with an error in classifying the non-injured group. When assessing performance between the CW and CM models, the CW has a lower error % of injury classification and overall model misclassification making it more favorable to use. Due to the small sample sizes of the catapult and strength models, the synthetic data was created on a less diverse sample set making this type of model susceptible to overfitting. While the catapult weekly and monthly models also have relatively low injury events, we were able to predict the 70 and 80 injury data points used in these models with low error % despite greater variability amongst the injured class. If we compare weekly and monthly models, the CSW and CSM perform better, however, it is important to take into consideration the increased sample size of the injury class in the CW and CM models.

We have demonstrated the ability to predict adductor strain in an MLS cohort provided that the entire data set is utilized and the influencing variables mentioned are included. When we performed SMOTE on the entire dataset and then proceeded with an 80% train and 20% test split, the random forest model successfully identified a subset of predictive valuables for adductor injury in an MLS cohort. As SMOTE interpolates from injury data points to create synthetic injury events, the model would likely not be successful in predicting injuries that had differing characteristics from the original injuries. While the models performed well, it is important to acknowledge the potential for overfitting, however, internal validation through bootstrapping was conducted to obtain *p*-values of variable importance for each predictive input variable. This emphasizes the need for further validation and greater sample sizes when utilizing this information for prediction protocols in a new cohort. Furthermore, with models developed from a subset of the data, we were unsuccessful in predicting new data for a novel season (i.e., 2022 season). This is likely due to the small sample size as we only had two injury occurrences in 2022 and currently alludes to lack of predictive robustness.

As mentioned by Bullock et al., there are shortcomings to the current models that have been utilized to predict sports musculoskeletal injuries [10]. By using the RF and LR methodology, we were able to address continuous and categorical variables appropriately and not assume linearity to give a better representation of the variables assessed.

### 4.1. Considerations and Future Objectives

While we have demonstrated the utility of RF and SMOTE to predict injury in a single cohort, there are limitations that should be taken into consideration. The number of non-injury data points recorded within the years of data collection greatly outweighed the number of injured data points collected. Due to this, the utilization of SMOTE techniques to create synthetic instances of injury was needed. When generating synthetic data, it is important to note that instances of future injury could be missed especially if the athlete exhibits different features compared to the current cohort. With the small injury sample size collected during this time frame, application to an injury prevention plan will need further validation and verification.

This model was built from a generalized adductor strain injury set. This means that the adductor strains mentioned could have occurred in any of the six notable adductor muscles. This generalization was needed due to the sample size of the injury. In the future, with a greater injured sample size, it would be advantageous to create predictive models for specific muscles within the adductor group. Each adductor muscle’s unique combination of primary and secondary functions as well as location and structure could influence injury risk. In addition, these techniques could be utilized to predict other common injuries in sports. While utilizing performance variables and past injury history is important in efforts to predict injury, further understanding of a person’s physiological predispositions, internal load measures, and muscle integrity could provide further information for identifying risk of injury. Soft tissue and non-contact injuries have the potential to be addressed early through training protocols and knowledge of a player’s predispositions such as past injury and position type. This information can give practitioners insight into appropriate training and injury mitigation for each of their athletes. For this study, we assessed a single team within the MLS. While this created a more consistent environment based on training and medical protocols, it should be noted that this could influence the model based on this specific controlled environment. For future application, practitioners and researchers should assess model performance in their own club setting.

### 4.2. Clinical Implications

We have combined RF and SMOTE techniques to identify which common game and performance metrics influence adductor injury risk in a cohort of MLS soccer players. The most common predictors of adductor injury include COD efforts, max hamstring force, ad/abductor max force ratio, position played, and prior injury. Future studies should use a larger sample size, with models applied to unique cohorts and muscle types, prior to the development of injury prevention protocols.

## Figures and Tables

**Table 1:** Collected Model Features and Corresponding Definitions.

Position, Injury, and Game Features	
P_for_	Forward
P_mid_	Midfielder
P_def_	Defender
P_gk_	Goalkeeper
G_num_	Number of games played
G_min_	Total minutes played in games
PI_never_	Record of previous adductor injury
PI_0–2_	0 to 2 months since previous adductor injury
PI_12+_	Over 12 months since a previous adductor injury
Adductor, Abductor, and Hamstring Strength Features	
Ab_maxf_	Abduction maximum force on nondominant leg
Ad_maxf_	Adduction maximum force on dominant leg
Ham_maxf_	Hamstring maximum force on dominant leg
Ab_maximb_	Abduction maximum force interlimb imbalance
Ad_maximb_	Adduction maximum force interlimb imbalance
Ham_maximb_	Hamstring maximum force interlimb imbalance
Ratio_adab_	Ratio of adductor over abductor dominant maximum force
IMU/GPS Features	
Accel_eff_	IMA acceleration efforts
Accel2_eff_	2nd generation acceleration efforts
COD_eff_	COD efforts toward nondominant side
EWMA_accel_	EWMA of acceleration effort
EWMA_accel2_	EWMA of 2nd generation acceleration efforts
EWMA_decel_	EWMA of IMA deceleration efforts
EWMA_cod_	EWMA of COD efforts towards nondominant side

**Table 2:** Model Datasets Pre Balancing (PB) and Post Balancing (POB).

	# Athletes	# Injured Athletes	# Injuries PB	# Injuries POB	# Non-Injuries PB	# Non-Injuries POB
CW (2019–2022)	53	7	8	80	2839	240
CM (2019–2022)	53	6	7	70	733	210
CSW (2021–2022)	37	5	5	50	1165	150
CSM (2021–2022)	37	4	4	40	292	120

**Table 3:** Results of the CW and CM RF and LR. Significant predictors are shaded gray and injury risk % can be computed as 1—odds ratio (OR).

CW Model			
Predictor Variable Name	VIMP	OR	*p*-Value
Accel_eff_	0.030	1.011	0.284
COD_eff_	0.035	1.041	0.289
EWMA_accel_	0.040	1.082	0.253
EWMA_cod_	0.050	1.112	0.096
EWMA_decel_	0.035	1.067	0.274
P_for_	0.096	4.182	0.152
P_mid_	0.052	1.118	0.094
P_def_	0.052	0.176	*p* < 0.05
P_gk_	0.007	1.179	0.382
G_num_	0.037	1.444	0.343
G_min_	0.034	1.004	0.273
PI_never_	0.099	0.193	*p* < 0.05
PI_0–2_	0.090	28.437	0.317
PI_12+_	0.130	4.288	0.491
CM Model			
Predictor Variable Name	VIMP	OR	*p*-Value
Accel_eff_	0.037	1.075	0.155
COD_eff_	0.028	1.057	*p* < 0.05
EWMA_accel_	0.052	1.193	0.160
EWMA_cod_	0.019	1.083	0.428
EWMA_decel_	0.028	1.073	0.317
P_for_	0.062	6.363	0.333
P_mid_	0.011	0.663	0.617
P_def_	0.009	*	0.198
P_gk_	0.023	2.029	0.250
G_num_	0.032	1.422	0.183
G_min_	0.025	1.002	0.370
PI_never_	0.110	0.094	*p* < 0.05
PI_0–2_	0.144	104.234	0.098
PI_12+_	0.114	7.253	0.144

Note: * signifies the non-convergence of the logistic regression.

**Table 4:** Results of the CSW and CSM RF and LR. Significant predictors are shaded gray and injury risk % can be computed as 1—odds ratio (OR).

CSW Model			
Predictor Variable Name	VIMP	OR	*p*-Value
Accel_eff_	0.016	0.944	0.253
Accel2_eff_	0.015	1.001	0.405
COD_eff_	0.075	1.004	*p* < 0.05
EWMA_accel_	0.009	0.923	0.413
EWMA_accel2_	0.022	1.009	0.091
EWMA_cod_	0.041	1.141	0.101
EWMA_decel_	0.019	0.458	0.317
P_for_	0.227	9.173	*p* < 0.05
P_mid_	0.011	0.363	*p* < 0.05
P_def_	0.019	0.458	*p* < 0.05
P_gk_	0.001	*	0.317
G_num_	0.006	1.238	0.708
G_min_	0.011	1.000	0.359
PI_never_	0.012	0.833	0.230
PI_0–2_	0.094	14.866	0.228
PI_12+_	0.000	*	1.000
Ab_maxf_	0.055	1.001	*p* < 0.05
Ad_maxf_	0.036	1.002	*p* < 0.05
Ham_maxf_	0.043	0.994	*p* < 0.05
Ab_maximb_	0.064	1.039	0.183
Ad_maximb_	0.086	1.014	0.051
Ham_maximb_	0.033	1.034	0.151
Ratio_adab_	0.029	0.138	*p* < 0.05
CSM Model			
Predictor Variable Name	VIMP	OR	*p*-Value
Accel_eff_	0.011	0.961	0.359
Accel2_eff_	0.053	1.053	*p* < 0.05
COD_eff_	0.006	1.040	0.391
EWMA_accel_	0.008	0.970	0.568
EWMA_accel2_	0.014	1.063	0.484
EWMA_cod_	0.019	1.101	0.144
EWMA_decel_	0.008	0.843	0.374
P_for_	0.186	19.998	*p* < 0.05
P_mid_	0.005	0.680	0.405
P_def_	0.005	*	0.096
P_gk_	0.000	*	1.000
G_num_	0.045	1.547	0.061
G_min_	0.019	1.002	0.084
PI_never_	0.016	0.458	*p* < 0.05
PI_0–2_	0.050	9.469	0.389
PI_12+_	0.000	*	1.000
Ab_maxf_	0.031	1.014	0.500
Ad_maxf_	0.011	1.006	0.563
Ham_maxf_	0.022	0.994	*p* < 0.05
Ab_maximb_	0.061	1.023	0.276
Ad_maximb_	0.033	1.021	0.509
Ham_maximb_	0.015	1.006	0.096
Ratio_adab_	0.037	16.956	*p* < 0.05

Note: * signifies the non-convergence of the logistic regression.

**Table 5:** Confusion matrix results of all models from RF.

	Error of Injured (%)	Error of Non-Injured (%)	Total Model Error (%)
CW (2019–2022)	8.75	0.83	2.81
CM (2019–2022)	11.43	0.48	3.21
CSW (2021–2022)	6.00	0.00	1.50
CSM (2021–2022)	5.00	0.83	1.88

## Data Availability

Restrictions apply to the availability of these data. Data permissions are needed by the MLS M-Marc Medical committee and are not available for public sharing.

## Abbreviations

The following abbreviations are used in this manuscript:

MLS	Major League Soccer
RF	Random Forest
SMOTE	Synthetic Minority Oversampling
RUS	Random Undersampling
VIMP	Variable Importance
OR	Odds Ratios
AUC	Area under the receiver operating curve
IMU	Inertial Measurement Unit
IMA	Inertial Movement Analysis
GPS	Global Positioning System
COD	Change of Direction
MOI	Mechanism of Injury
EWMA	Exponential Weighted Moving Average
LR	Logistic Regression
PB	Pre Balancing
POB	Post Balancing
CW	Catapult Weekly
CM	Catapult Monthly
CSW	Catapult Strength Weekly
CSM	Catapult Strength Monthly
